# Supplementary material for: Insights into the ecological roles and evolution of methyl-coenzyme M reductase-containing hot spring Archaea
Source: Nat Commun. 2019 Oct 8;10:4574. doi: 10.1038/s41467-019-12574-y (PMC6783470; doi:10.1038/s41467-019-12574-y)
Supplement: Supplementary file 1 — Supplementary Information [file 41467_2019_12574_MOESM1_ESM.pdf]

Supplementary Information

Insights into the ecological roles and evolution of methyl-coenzyme M reductase containing hot spring Archaea

Hua et al.

Supplementary Figures

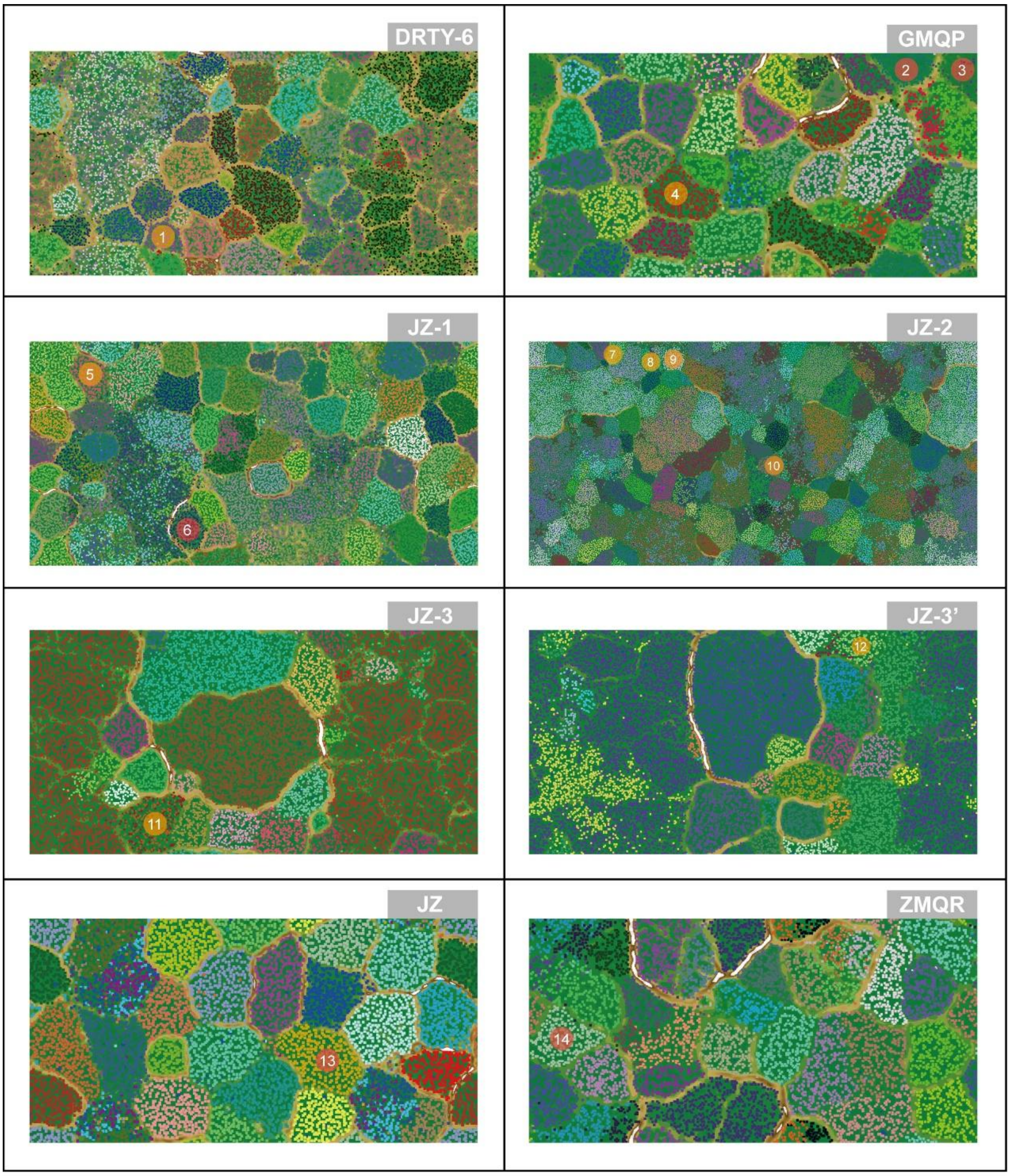

1. DRTY-6 bin_144
2. GMQP bin_32
3. GMQP bin_44
4. GMQP bin_37
5. JZ-1 bin_66
6. JZ-1 bin_103
7. JZ-2 bin_199
8. JZ-2 bin_200
9. JZ-2 bin_220
10. JZ-2 bin_168
11. JZ-3 bin_106
12. JZ-3 bin_107
13. JZ bin_38
14. ZMQR bin_18

Supplementary Figure 1. Visualization of metagenomic-assembled bins in this study using ESOM (Emergent Self-Organizing map)[1]. The sampling sites include DRTY-6, GMQP, JZ-1, JZ-2, JZ-3, JZ and ZMQR as indicated in each box. Bins in number 11 and 12 (JZ-3 bin_106 and JZ-3 bin_107) are collected from same site (JZ-3) but different times. Here, we used a single quote to distinguish them.

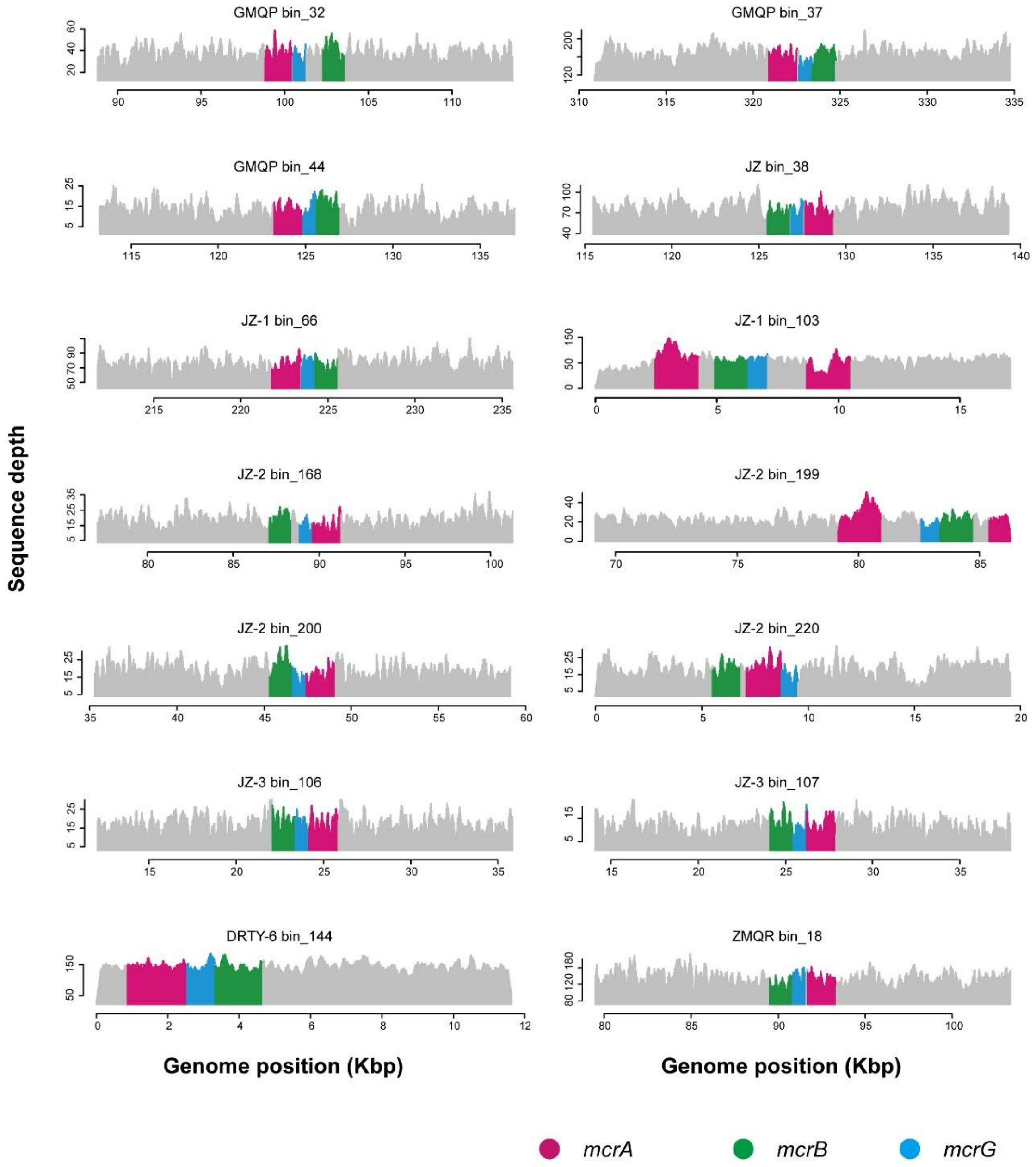

Supplementary Figure 2. The histograms show the coverage information of the *mcrABG*-contained scaffolds. Besides the *mcrABG* genes, only the first and last 5000 bp before and after the *mcrABG* region were shown for those longer scaffolds. Otherwise, coverage information for the whole scaffolds were shown.

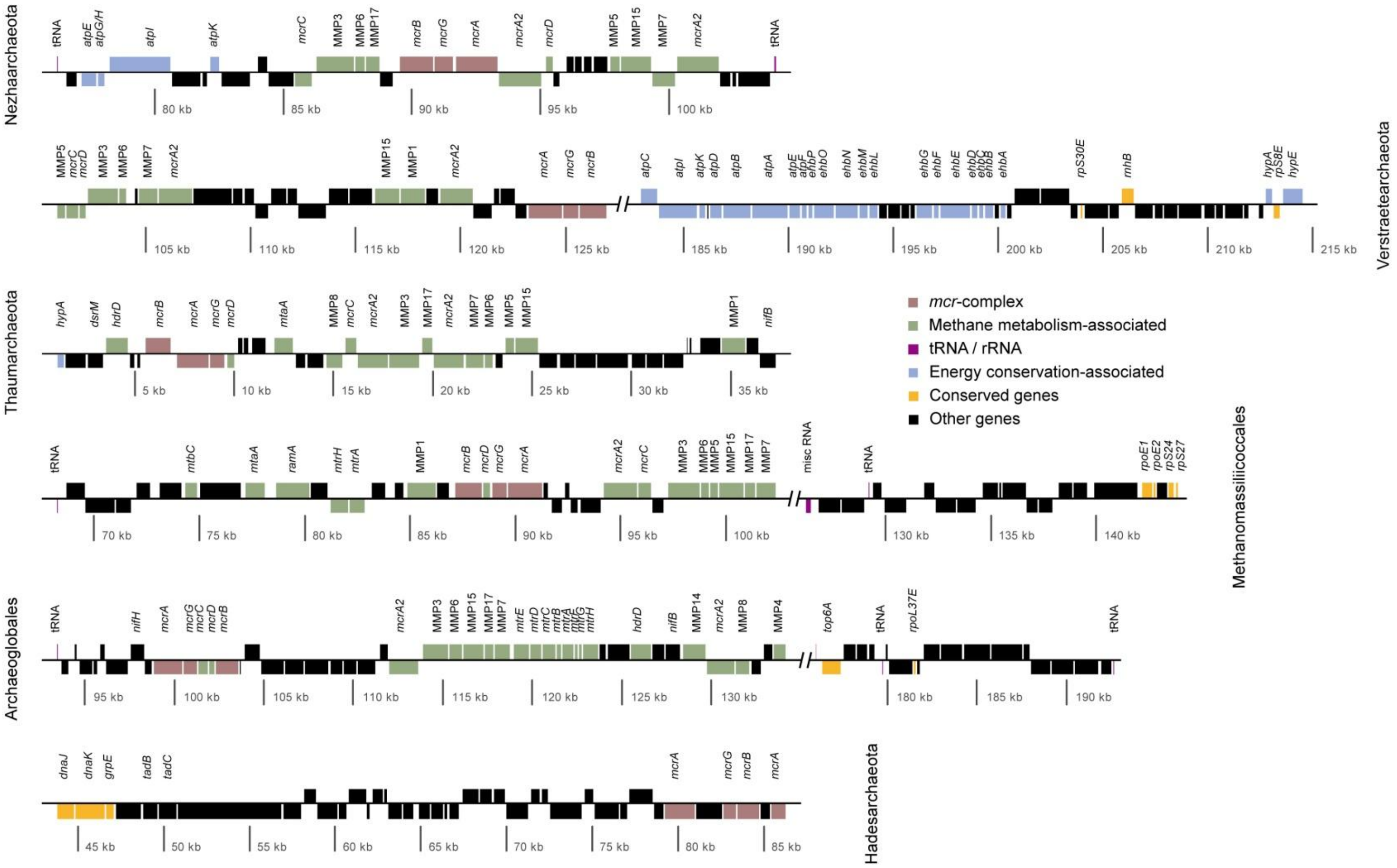

Supplementary Figure 3. Schematic representation of gene clusters of *mcr*-complex and genes nearby. Different colors indicate different type of genes, including the McrABG complex (red), methane/alkane metabolism-associated (green), tRNAs/rRNAs (pink), energy conservation (blue), conserved marker genes (orange, such as, ribosomal proteins) and other genes (black). Rectangulars above (+) /below (-) the lines indicate the orietation of genes located in the scaffolds. Abbreviations: MMP, methanogenesis marker protein.

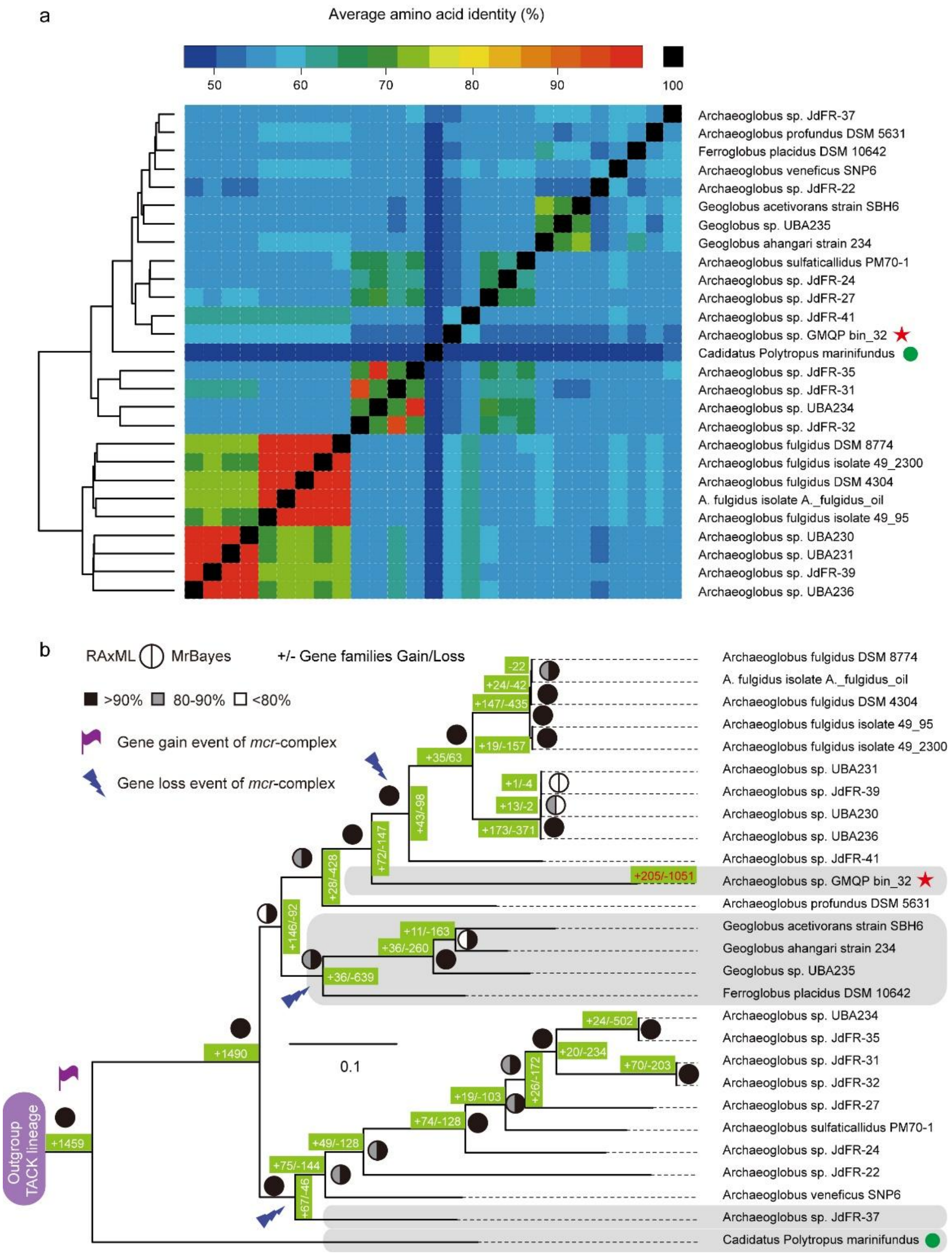

Supplementary Figure 4. Evolutionary history inference of *Archaeoglobales* lineage. a) All the sequenced genomes belonged to order *Archaeoglobales* were downloaded from NCBI public database (Supplementary Data 6). Hierarchical clustering heatmap based on average amino acids identity for each genome pair. b) Ancestral genome content was reconstructed[2] for current available *Archaeoglobales*-belonged genomes. Both the Bayesian tree and maximal-likelihood tree generated from MrBayes[3] (v3.2.6) and IQ-TREE[4] (v1.6.10, ultrafast bootstrap = 1000) show the same phylogenetic topology. The numbers of gain and loss events were marked at each lineage of the tree. “+”s represent gain events and “-”s represent loss events. Genomes shaded in grey boxes are non-SRMs (non-Sulfate-reducing microorganisms). Red star indicates the MAG in this study. Green circle represents a MAG with McrABG complex from other study. Flag / lighting symbols indicate the gene gain / loss of *mcr*-complex.

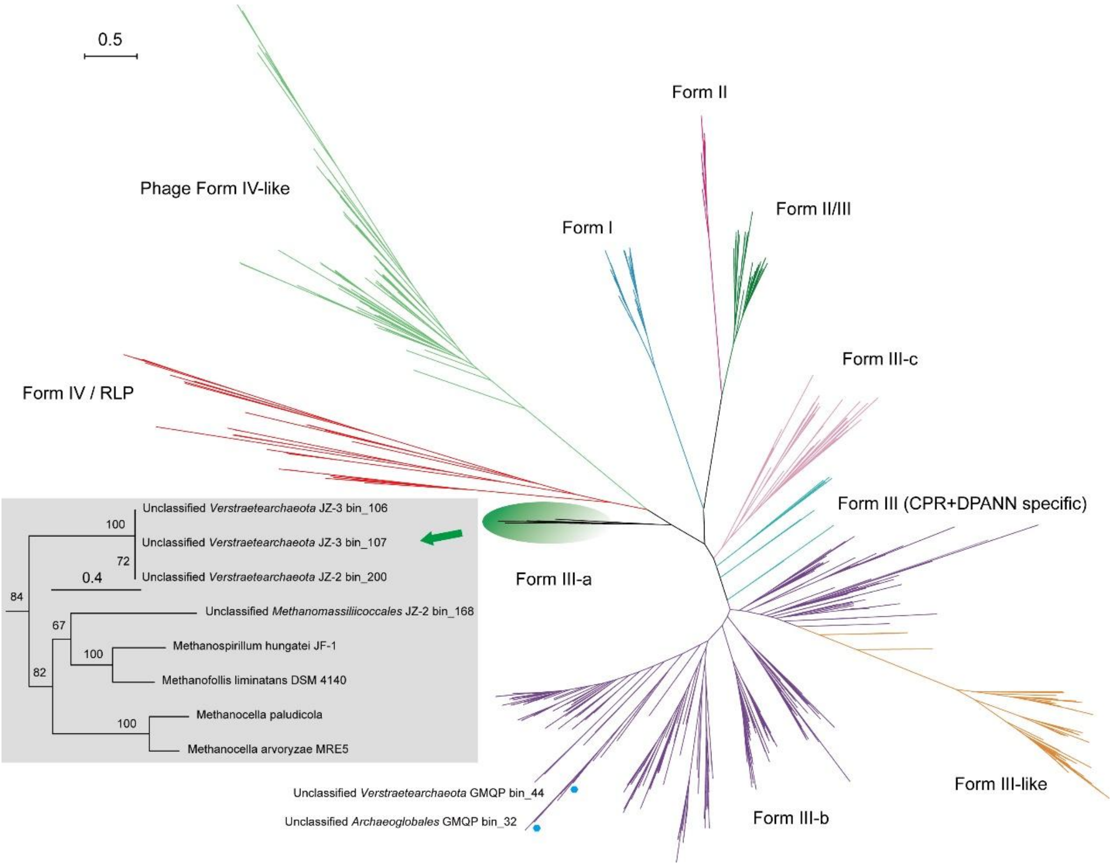

Supplementary Figure 5. Phylogenetic trees of RuBisCOs and associated proteins. Reference amino acid sequences from the tree are from a recent study[5] and aligned using MAFFT[6]. TrimAl[7] was used to eliminate divergent regions. Phylogenetic tree was constructed using RaxML[8] (v7.2.7, bootstrap = 100) under the PROTJTTGAMMAI model with 100 replicates.

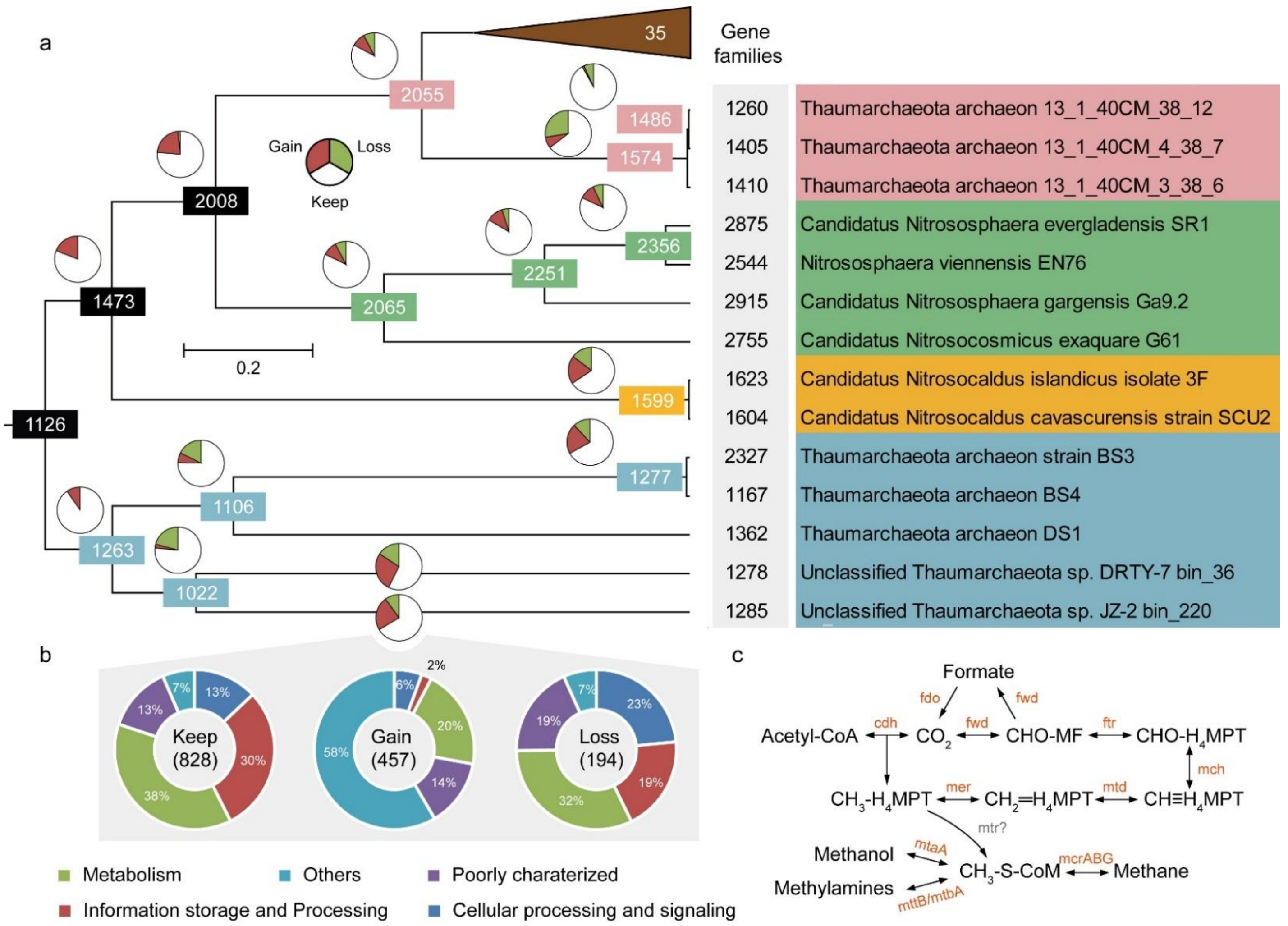

Supplementary Figure 6. Evolutionary history inference of Thaumarchaeota phylum. a) Ancestral genome content was reconstructed for current available Thaumarchaeota genomes from public databases using COUNT software[2]. The Bayesian tree topology was determined by MrBayes[3]. All nodes seen above are in high confidences with posterior possibililties equal to 1. Different lineages including pSL12 (potential), HWCGIII (hot water crenchaeotic group), Group I.1b, unknown and group I.1a groups are shaded in blue, orange, green, pink and brown colors from bottom to top. See Supplementary Data 6 for detailed group information of each genome. The pie plots show the gene gain and loss events at all nodes. b) The pie plots show the number of genes inherited, gained and lost genes by COG categories for MAG JZ-2 bin_220 in this study. C). The pathway shows the gained genes related to methanogenesis pathway. All gained genes are colored in orange.

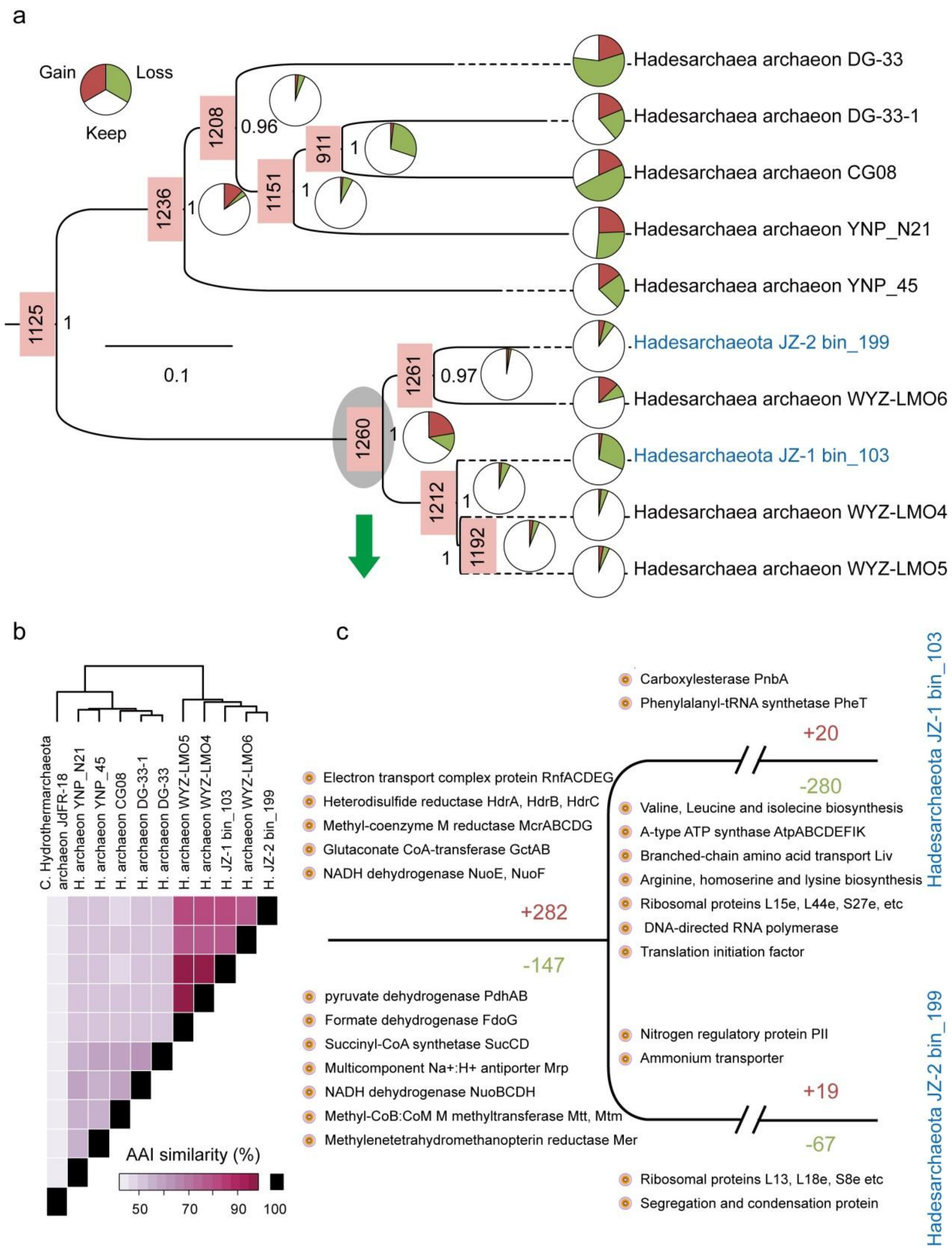

Supplementary Figure 7. Evolutionary history inference of *Hadesarchaeota* lineage. a) All the sequenced genomes belonged to Hadesarchaeota were downloaded from NCBI public database (Supplementary Data 6). The Bayesian tree topology was determined by MrBayes[3]. Ancestral genome content was reconstructed using COUNT[2] based on the genomes available. b) Hierarchical clustering heatmap based on average amino acids identity for each genome pair. c) The main gene gain and loss events for MAGs at the node of JZ-1 bin_103 and JZ-2 bin_199 and their ancestor are shown as listed.

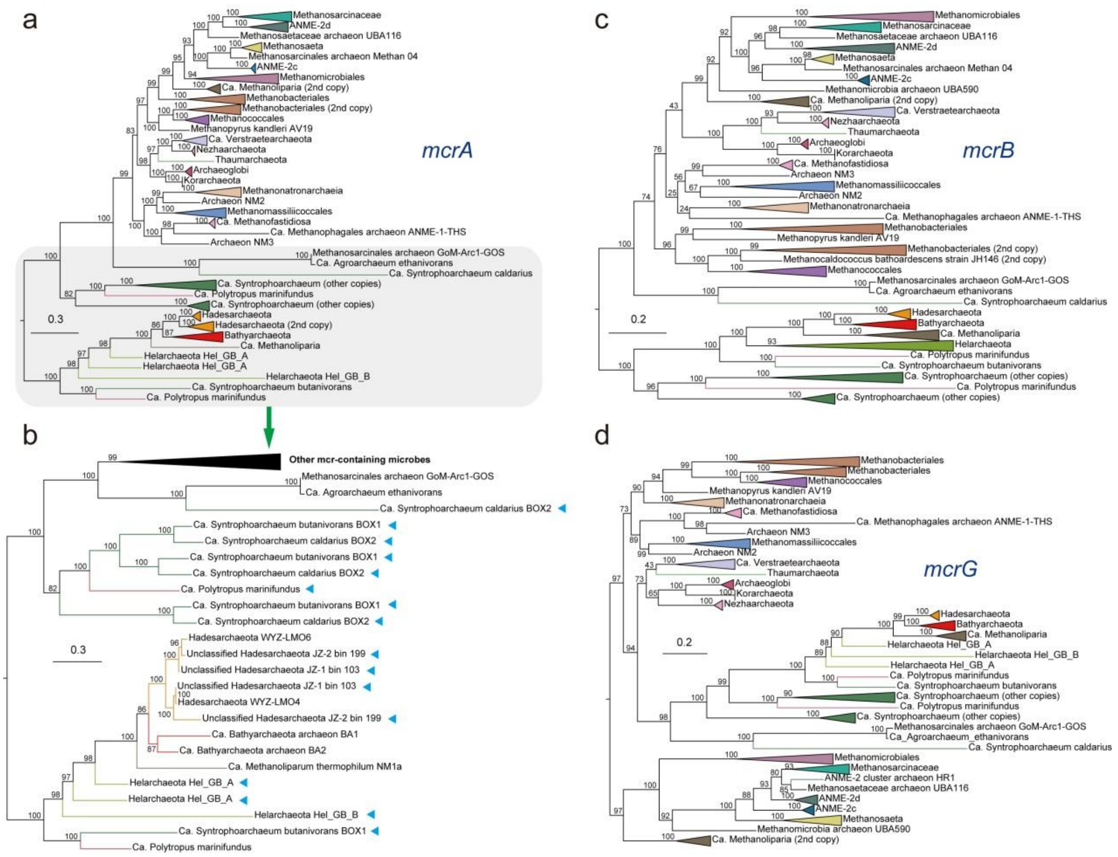

Supplementary Figure 8. Phylogenies of subunits of the methyl-coenzyme M reductase complex. Amino acid sequences of *mcrABG* genes were searched against 14 genomes in this study and reference genomes (Supplementary Data 5) downloaded from public databases. Phylogenetic trees of **a**) *mcrA*, **b**) zoomed-in of the alkanotroph lineage of *mcrA* gene, **c**) *mcrB* and **d**) *mcrG* genes were inferred using IQ-TREE[4] (v1.6.10, ultrafast bootstrap = 1000). See Methods for detailed settings of parameters. Only ultrafast bootstrap values >70% were shown on nodes. All bootstrap values on **b**) are > 80%. Blue triangles in b) show multiple copies of corresponding genomes/MAGs.

Supplementary Tables

Supplementary Table 1. Physical and chemical characteristics of the six hot spring sampling sites.

Sample	North	South	Temp	pH	TOC	DOC	Cl^-	NO_2^-	NO_3^-	NH_4^+	SO_4^{2-}	Na^+	K^+	Mg^{2+}	Ca^{2+}
JZ-1	$25^0$26'28"	$98^0$27'36"	86.5	7.3	22	32	176	29	3.8	4.7	28	430	34	6.4	18
JZ-2	$25^0$26'28"	$98^0$27'36"	63	7.6											
JZ-3	$25^0$26'28"	$98^0$27'36"	71	7.6											
JZ	$25^0$26'28"	$98^0$27'36"	75	6.5	6.8	22	141	21	3.5	3.4	19	300	30	4.0	17
GMQP	$24^0$57'3"	$98^0$26'10"	87	9.6	0.33	39	655	25	3.9	6.6	41	772	71	0.50	n.a.
ZMQR	$24^0$57'4"	$98^0$26'10"	98	9.5											
DRTY-6	$24^0$57'14"	$98^0$26'18"	60	6.0	98	577	6.8	11	5.2	9.9	825	14	43	46	267

All values are in mg L^{-1}, except pH (in standard units), Temperature (in ^oC) and TOC (in mg g^{-1}).

Abbreviations: TOC, total organic carbon; DOC, dissolved organic carbon.

Supplementary Table 2. GC content comparison between *mcr*-complex and corresponding genomes.

MAGs		GC content (%)	
		Genome*	*mcr*-complex†
Nezha-	JZ bin_38	43.7 ±0.18	44.6
	JZ-1 bin_66	43.6 ±0.04	43.6
	GMQP bin_37	45.1 ±0.05	45.2
	ZMQR bin_18	45.7 ±0.32	45.4
Verstraete-	JZ-2 bin_200	47.2 ±0.34	44.9
	JZ-3 bin_106	47.1 ±0.35	45.1
	JZ-3 bin_107	46 ±0.43	46.2
	GMQP bin_44	27.8 ±0.43	29
	DRTY-6 bin_144	46.6 ±0.34	46.3
Eury-	JZ-1 bin_103	56.2 ±0.27	53.1
	JZ-2 bin_168	43.5 ±0.46	43.1
	JZ-2 bin_199	62.6 ±0.33	61.5
	GMQP bin_32	41.1 ±0.27	41.3
Thaum-	JZ-2 bin_220	38.9 ±0.15	40.1

* GC content for genome was calculated as Mean ± SE of all scaffolds in corresponding MAGs.

† GC content for *mcr*-complex was computed as number of Gs and Cs in the *mcr*-containing scaffold.

Abbreviations: GC, guanine-cytosine; Nezha-, *Nezhaarchaeota*; Verstraete-, *Verstraetearchaeota*; Eury-, *Euryarchaeota*; Thaum-, *Thaumarchaeota*.

Supplementary Table 3. Taxonomy of selected metagenomic bins according to Genome Taxonomy Database and proposed names.

Group	Metagenomic bin	Phylum	Class	Order	Family	Genus	Species	Type material
Nezha-	JZ bin_38	Nezhaarchaeota	Nezhaarchaea	Nezhaarchaeales	**Methanohydrogenotrophicaceae***	**Methanohydrogenotrophicum***	**pristinum***	Ga0180368
	JZ-1 bin_66	Nezhaarchaeota	Nezhaarchaea	Nezhaarchaeales	**Methanohydrogenotrophicaceae***	**Methanohydrogenotrophicum***	**pristinum***	Ga0263245
	GMQP bin_37	Nezhaarchaeota	Nezhaarchaea	Nezhaarchaeales	**Methanohydrogenotrophicaceae***	**Methanogeoarchaeum***	**hydrogenovorans***	Ga0263257
	ZMQR bin_18	Nezhaarchaeota	Nezhaarchaea	Nezhaarchaeales	**Methanohydrogenotrophicaceae***	**Methanogeoarchaeum***	**hydrogenovorans***	Ga0263256
Verstraete-	JZ-2 bin_200	Crenarchaeota	Methanomethylicia	**Methanomethylovorales***	**Methanomethylovoraceae***	**Methanomethylovorus***	**thermophilus***	Ga0263249
	JZ-3 bin_106	Crenarchaeota	Methanomethylicia	**Methanomethylovorales***	**Methanomethylovoraceae***	**Methanomethylovorus***	**thermophilus***	Ga0263252
	JZ-3 bin_107	Crenarchaeota	Methanomethylicia	**Methanomethylovorales***	**Methanomethylovoraceae***	**Methanomethylovorus***	**thermophilus***	Ga0263255
	GMQP bin_44	Crenarchaeota	Methanomethylicia	**Methanomethyloarchaeales***	**Methanomethyloarchaeceae***	**Methanomethyloarchaeum***	**antiquum***	Ga0263253
	DRTY-6 bin_144	Crenarchaeota	Methanomethylicia	**Methanomethylovorales***	**Methanomethylovoraceae***	**Methanomethylovorus***	**thermophilus***	Ga0263254
Eury-	JZ-1 bin_103	Hadesarchaeota*	**Hadesarchaea***	**Hadesarchaeales***	**Hadesarchaeceae***	**Methanourarchaeum***	**thermotelluricum***	Ga0263246
	JZ-2 bin_168	Thermoplasmatota	Methanomassiliicocci*	Methanomassiliicoccales	**Methanomixtatrophicaceae***	**Methanomixtatrophicum***	**sinensis***	Ga0263247
	JZ-2 bin_199	Hadesarchaeota*	**Hadesarchaea***	**Hadesarchaeales***	**Hadesarchaeceae***	**Hadesarchaeum***	**tengchongensis***	Ga0263248
	GMQP bin_32	Halobacteraeota	Archaeoglobi	Archaeoglobales	Archaeoglobaceae	**Methanoproducendum***	**senex***	Ga0263258
Thaum-	JZ-2 bin_220	Crenarchaeota	Nitrososphaeria	Nitrososphaerales	**Methylarchaceae***	**Methylarchaeum***	**tengchongensis***	Ga0263250

* Proposals for new nomenclature are indicated with asterisk and in bold. Items shaded with yellow indicate changes compared to previous nomenclature.

References

1. Dick, G. J. et al. Community-wide analysis of microbial genome sequence signatures. *Genome Biol*. **10**, R85 (2009).
2. Csűös, M. Count: evolutionary analysis of phylogenetic profiles with parsimony and likelihood. *Bioinformatics* **26**, 1910-1912 (2010).
3. Ronquist, F., et al. MrBayes 3.2: efficient Bayesian phylogenetic inference and model choice across a large model space. *Syst. Biol.* **61**, 539-542 (2012).
4. Nguyen, L. T., Schmidt, H. A., von Haeseler, A., & Minh, B. Q. IQ-TREE: a fast and effective stochastic algorithm for estimating maximum-likelihood phylogenies. *Mo. Biol. Evol.* **32**, 268-274 (2015).
5. Jaffe, A. L., Castelle, C. J., Dupont, C. L., & Banfield, J. F. Lateral gene transfer shapes the distribution of RuBisCO among Candidate Phyla Radiation bacteria and DPANN archaea. bioRxiv 386292 (2018).
6. Katoh, K., & Standley, D. M. MAFFT multiple sequence alignment software version 7: improvements in performance and usability. *Mol. Biol. Evol.* **30**, 772-780 (2013).
7. Capella-Gutiérrez, S., Silla-Martínez, J. M., & Gabaldón, T. trimAl: a tool for automated alignment trimming in large-scale phylogenetic analyses. *Bioinformatics* **25**, 1972-1973 (2009).
8. Stamatakis, A. RAxML-VI-HPC: maximum likelihood-based phylogenetic analyses with thousands of taxa and mixed models. *Bioinformatics* **22**, 2688-2690 (2006).
