# Supplementary material for: Insights into the ecological roles and evolution of methyl-coenzyme M reductase-containing hot spring Archaea
Source: Nat Commun. 2019 Oct 8;10:4574. doi: 10.1038/s41467-019-12574-y (PMC6783470; doi:10.1038/s41467-019-12574-y)
Supplement: Supplementary file 3 — Reporting Summary [file 41467_2019_12574_MOESM3_ESM.pdf]

nature research

Corresponding author(s): Wen-Jun Li, Tong Zhang, Gene W Tyson

Last updated by author(s): Aug 27th, 2019

Reporting Summary

Nature Research wishes to improve the reproducibility of the work that we publish. This form provides structure for consistency and transparency in reporting. For further information on Nature Research policies, see Authors & Referees and the Editorial Policy Checklist.

Statistics

For all statistical analyses, confirm that the following items are present in the figure legend, table legend, main text, or Methods section.

n/a	Confirmed	
☐	☒	The exact sample size (*n*) for each experimental group/condition, given as a discrete number and unit of measurement
☐	☒	A statement on whether measurements were taken from distinct samples or whether the same sample was measured repeatedly
☐	☒	The statistical test(s) used AND whether they are one- or two-sided<br>*Only common tests should be described solely by name; describe more complex techniques in the Methods section.*
☐	☒	A description of all covariates tested
☐	☒	A description of any assumptions or corrections, such as tests of normality and adjustment for multiple comparisons
☐	☒	A full description of the statistical parameters including central tendency (e.g. means) or other basic estimates (e.g. regression coefficient) AND variation (e.g. standard deviation) or associated estimates of uncertainty (e.g. confidence intervals)
☐	☒	For null hypothesis testing, the test statistic (e.g. *F*, *t*, *r*) with confidence intervals, effect sizes, degrees of freedom and *P* value noted<br>*Give P values as exact values whenever suitable.*
☐	☒	For Bayesian analysis, information on the choice of priors and Markov chain Monte Carlo settings
☐	☒	For hierarchical and complex designs, identification of the appropriate level for tests and full reporting of outcomes
☐	☒	Estimates of effect sizes (e.g. Cohen's *d*, Pearson's *r*), indicating how they were calculated

Our web collection on statistics for biologists contains articles on many of the points above.

Software and code

Policy information about availability of computer code

Data collection	No software was used for data collection. All reference genomes and corresponding parameters were downloaded and collected from public databases or published literatures manually.
Data analysis	Open source tools:<br>1. Metagenomic sequence assembly: SPAdes (v3.9.0)<br>2. Scaffold gaps remover: GapCloser (v1.12)<br>3. Sequences mapping: bbmap (v35.85)<br>4. .bam file parser: samtools (v1.3.1)<br>5. Genome binning: Metabat (v2.12.1)<br>6. MAGs visualization: ESOM (v1.1)<br>7. Genome bins quality evaluation: CheckM (v1.0.5)<br>8. Gene prediction: Prodigal (v2.6.3)<br>9. Local alignment searching: DIAMOND (v0.9.22.123) and HMMer (v3.1b2)<br>10. Marker gene recruitment: AMPHORA (v2)<br>11. Sequence alignment: MUSCLE (v3.8.31)<br>12. Poorly alignment regions remover: TrimAl (v1.4.rev22)<br>13. Phylogenetic tree construction: IQ-TREE (v1.6.10), RAxML (v7.2.7)<br>14. Tree visualization: iTOL (v3)<br>15. Ancestral amino acid sequence reconstruction: PAML (v4.9h)<br>16. Homologous genes clustering: MCL (v14-137)<br>17. Evolutionary history inference: COUNT (v9.1106)

For manuscripts utilizing custom algorithms or software that are central to the research but not yet described in published literature, software must be made available to editors/reviewers. We strongly encourage code deposition in a community repository (e.g. GitHub). See the Nature Research guidelines for submitting code & software for further information.

Data

Policy information about availability of data

All manuscripts must include a data availability statement. This statement should provide the following information, where applicable:

- Accession codes, unique identifiers, or web links for publicly available datasets
- A list of figures that have associated raw data
- A description of any restrictions on data availability

The 14 near-complete archaeal genomes are publicly available in the JGI IMG-MER under the Study ID Gs0127627 and WGS accessions Ga0180368 (Unclassified Crenarchaeota JZ bin_38), Ga0263245 (Unclassified Crenarchaeota JZ-1 bin_66), Ga0263257 (Unclassified Crenarchaeota GMQP bin_37), Ga0263256 (Unclassified Crenarchaeota ZMQR bin_18), Ga0263254 (Unclassified Verstraetearchaeota DRTY-6 bin_144), Ga0263253 (Unclassified Verstraetearchaeota GMQP bin_44), Ga0263249 (Unclassified Verstraetearchaeota JZ-2 bin_200), Ga0263252 (Unclassified Verstraetearchaeota JZ-3 bin_106), Ga0263255 (Unclassified Verstraetearchaeota JZ-3 bin_107), Ga0263246 (Unclassified Hadesarchaeota JZ-1 bin_103), Ga0263248 (Unclassified Hadesarchaeota JZ-2 bin_199), Ga0263247 (Unclassified Methanomassiliicoccales JZ-2 bin_168), Ga0263258 (Unclassified Archaeoglobales GMQP bin_32) and Ga0263250 (Unclassified Crenarchaeota JZ-2 bin_220). The datasets generated during and/or analyzed during the current study are available from the corresponding author on reasonable request.

Field-specific reporting

Please select the one below that is the best fit for your research. If you are not sure, read the appropriate sections before making your selection.

☐ Life sciences ☐ Behavioural & social sciences ☒ Ecological, evolutionary & environmental sciences

For a reference copy of the document with all sections, see nature.com/documents/nr-reporting-summary-flat.pdf

Ecological, evolutionary & environmental sciences study design

All studies must disclose on these points even when the disclosure is negative.

Study description	The main point of this study is to find novel microbes whom might be methanogens/methanotrophs/alkanotrophs. After the reconstruction of their genomic information, their metabolic potentials and evolutionary histories were inferred and revealed.
Research sample	Our research samples are collected from hot spring sediments in Tengchong County, Yunnan province, China. Those samples contain large quantity of bacterial and archaeal cells which are the main research objects.
Sampling strategy	Previous studies show that hot spring sediments could produce methane. So hot spring sediments samples were widely collected using sterile spatulas and spoons and stored in liquid nitrogen before transporting to the lab. The co-author Yangzhi Rao and Yuxian Li did the sample collection. Six samples from four different sites were identified to contain methanogens/methanotrophs/ alkanotrophs.
Data collection	Community genomic DNA was extracted from approximately 20 g of sediment material using PowerSoil DNA Isolation kit (MoBio). DNA concentrations of the extract and constructed libraries (with insert size of 350 bp) were measured with a Qubit fluorometer. Metagenomic sequence data for the two samples are generated using Illumina Hiseq 4000 instruments at Beijing Novogene Bioinformatics Technology Co., Ltd (Beijing, China). The amount of raw sequence data was ~30 Gbp (2×150bp) for each sample.
Timing and spatial scale	Samples were collected in March, 2017.
Data exclusions	No data was excluded.
Reproducibility	No replicates were conducted since it is not relevant.
Randomization	It is not relevant to our study since our study is to discover the novel organisms that might be methanogens/methanotrophs/ alkanotrophs. So only samples contain those microbes were kept.
Blinding	Mis-assembled scaffolds introduced by assemblers may result in the blinding. Also, bias might be existed during the genome binning step. To avoid this, we used ESOM to visualize genome bins to see if scaffolds in each bin clustered together. Besides, reads were mapped onto mcrABG-containing scaffolds to see the evenness among this and nearby region. Both results are perfect in our case.

Did the study involve field work? ☒ Yes ☐ No

Field work, collection and transport

Field conditions	Samples are from thermal habitats with temperature ranging from 60~98 °C and pH ranging from 6.0 to 9.6.
Location	Totally six metagenomic samples distributed in following four locations (pools): JZ (JinZe; 25.441E, 98.46N), GMQ (GuMingQuan; 24.951E, 98.436N), DRTY (DiReTiYan; 24.954E, 98.438N), and ZMQ (ZiMeiQuan; 24.954E, 98.436N) in this study were collected from thermal spring sediments in Tengchong in Yunnan (China) which located at the collision boundary between the India and Eurasia plates.
Access and import/export	Since these samples were collected by ourselves in China, so we no need special sampling permission and also our samples will

Access and import/export	not be imported or exported at all. Any, we will add one sentence during our revision process for this manuscript in the Acknowldegements as following: We also thank the entire staff from Yunnan Tengchong Volcano and Spa Tourist Attraction Development Corporation for strong support.
Disturbance	No disturbance.

Reporting for specific materials, systems and methods

We require information from authors about some types of materials, experimental systems and methods used in many studies. Here, indicate whether each material, system or method listed is relevant to your study. If you are not sure if a list item applies to your research, read the appropriate section before selecting a response.

Materials & experimental systems

n/a	Involved in the study
☒	☐ Antibodies
☒	☐ Eukaryotic cell lines
☒	☐ Palaeontology
☒	☐ Animals and other organisms
☒	☐ Human research participants
☒	☐ Clinical data

Methods

n/a	Involved in the study
☒	☐ ChIP-seq
☒	☐ Flow cytometry
☒	☐ MRI-based neuroimaging
