# Supplementary material for: Insights into the ecological roles and evolution of methyl-coenzyme M reductase-containing hot spring Archaea
Source: Nat Commun. 2019 Oct 8;10:4574. doi: 10.1038/s41467-019-12574-y (PMC6783470; doi:10.1038/s41467-019-12574-y)
Supplement: Supplementary file 4 — Description of Additional Supplementary Files [file 41467_2019_12574_MOESM4_ESM.pdf]

Description of Additional Supplementary Files

Supplementary Data 1: Maximum likelihood tree based on 122 concatenated conserved marker genes, basis for main text Figures 1, in newick format. See Methods for detailed phylogenetic tree reconstruction.
Supplementary Data 2: Maximum likelihood tree based on concatenated *mcrABG* genes, basis for main text Figures 3, in newick format. See Methods for detailed phylogenetic tree reconstruction.
Supplementary Data 3: List of genes and featured in the main text.
Supplementary Data 4: Detected gene gain and loss events at key nodes.
Supplementary Data 5: The genome characteristics of the selected 91 complete and draft genomes (downloaded from public databases) in this research which were used to construct the concatenated mcrABG gene tree.
Supplementary Data 6: Reference genomes downloaded from public databases for the comparative genomics.
